# Applications of mPCR testing reduced initial antibiotic use and duration of mechanical ventilation in virus-infected children with severe community-acquired pneumonia admitted to the PICU

**DOI:** 10.1007/s12519-022-00528-2

**Published:** 2022-03-29

**Authors:** Chao-Nan Fan, Bo-Liang Fang, Heng-Miao Gao, Ru-Bo Li, Guo-Yun Su, Yi-Yang Mao, Yu-Shan He, Yue Wang, Xiao-He Zhou, Liang-Ming Cai, Ye-Qing wang, Jennifer A. Blumenthal, Su-Yun Qian

**Affiliations:** 1 Pediatric Intensive Care Unit, Beijing Children’s Hospital, Capital Medical University, National Center for Children’s Health, No.56 Nan-Li-Shi Road, Beijing, 100045 China; 2 Division of Critical Care Medicine/Bader 6, Department of Anesthesiology, Critical Care and Pain Medicine, Boston Children’s Hospital and Harvard Medical School, 300 Longwood Avenue, Boston, MA 02115 USA

Community-acquired pneumonia (CAP) remains the leading cause of morbidity and mortality among children worldwide. It is critical for these patients to select and timely initiate appropriate empirical antimicrobial therapy against the causative pathogens [1]. However, conventional pathogen-detecting methods, such as culture and serology, have no prospect of altering empiric therapy owing to their time delay in obtaining results and to their lower detection rates [2]. Recent advances in molecular diagnostic assays, such as multiplex polymerase chain reaction PCR (mPCR) methods, have been used to detect multiple pathogens in CAP simultaneously within two hours and have dramatically improved the ability to diagnose respiratory pathogens [3]. However, whether this molecular diagnosis method can reduce the use of antibiotics and can improve prognosis in severe CAP children, especially those less than 5 years old, remains to be explored.

We conducted a retrospective cohort study enrolling 219 children with severe CAP in the pediatric intensive Care Unit (PICU) between 1 January 2016 and 31 December 2018, including 151 patients in the traditional testing group and 68 in the mPCR testing group. Respiratory samples from all patients were collected within 12 hours of admission to PICU. In the traditional testing group, routine microbiological methods were performed and children received empirical antimicrobial treatment. In the mPCR testing group, children underwent the mPCR testing within 12 hours of admission to PICU and selective use of antibiotics according to the rapid pathogen detection results.

About 77% (169/219) of patients were under 5 years; and there was no significant difference in the median age of these two groups. The gender composition was also similar. Other baseline clinical characteristics and laboratory results did not differ significantly between these two groups (Table 1).

**Table 1:** Patient demographics and clinical characteristics

Baseline characteristics	Traditional testing (*n* = 151)	mPCR testing (*n* = 68)	*P*
Age in year, median (IQR)	1.0 (0.5–5.2)	0.9 (0.4–5.0)	0.187
< 1 y, *n* (%)	78 (52%)	36 (53%)	0.86
> 5 y, *n* (%)	36 (24%)	16 (23%)	0.96
Male sex, *n* (%)	90 (60%)	39 (57%)	0.754
Days of fever before admission, median (IQR)	4 (2–6)	5 (2–8)	0.496
Mean (SD) heart rate (/min)	153 (21)	150 (27)	0.230
Mean (SD) respiratory rate (/min)	39 (10)	36 (11)	0.062
Laboratory values median, *n* (%)			
WBC > 10 × 10^9^/L	68 (45%)	29 (43%)	0.742
CRP > 8 mg/L	92 (60%)	38 (56%)	0.482
PCT > 0.25 ng/mL	88 (58%)	39 (57%)	0.897
Respiratory support before admission, *n* (%)			
Trachea intubation	34 (22%)	15 (22%)	0.94
Non-invasive ventilation	76 (50%)	33 (49%)	0.805
Nasal cannula	28 (19%)	13 (19%)	0.92
Complications, *n* (%)			
Heart failure	34 (23%)	10 (15%)	0.182
Respiratory failure	90 (60%)	42 (61%)	0.762
Acute renal failure	4 (3%)	4 (5%)	0.238
Liver dysfunction	15 (10%)	10 (15%)	0.304
Meningitis or encephalitis	21 (14%)	20 (29%)	0.006
MODS	3 (2%)	2 (3%)	0.662
Pleural fluid	23 (16%)	14 (21%)	0.338
PIM2 (%), median (IQR)	10 (3–20)	13 (2–24)	0.136
Main infiltrate pattern in chest X-ray at admission, *n* (%)			
Consolidation	62 (41%)	29 (43%)	0.971
Interstitial infiltrate	58 (38%)	27 (40%)	0.856
Mixed	29 (19%)	10 (15%)	0.421
Initial antibiotic use, *n* (%)			
Antibiotics before hospitalization	123 (82%)	53 (78%)	0.544
Any antibiotics before culture	140 (93%)	60 (89%)	0.276

*CRP* C-reaction protein, *IQR* interquartile range, *MODS* multiple organ dysfunction syndrome, *PCR* polymerase chain reaction, *PCT* procalcitonin, *PIM2* pediatric index of mortality 2, *WBC* white blood cell

The etiological identification rate in the traditional and mPCR testing groups were 72.4% and 77.8%, respectively, of which bacterial infections (42.9% vs. 47.1%), viral pathogens (51.0% vs. 55.9%) and *mycoplasma pneumonia* (15% vs. 17%) were determined. No significant differences were detected in the distribution of viral and bacterial pathogens between these two groups. In both the traditional and the mPCR testing groups, the most common bacteria found were *S. pneumoniae* (20% vs. 19%), *S. aureus* (14% vs. 21%), and *H. influenza* (18% vs. 13%). The most common viruses were respiratory syncytial virus (43% vs. 45%) and influenza (42% vs. 30%), respectively (Table 2). The detection rate of coronavirus was only 8% in the mPCR testing group, and coronavirus was not tested for the traditional testing methods.

**Table 2:** Microbiological yield in the study population

Etiological agents	No. patients with each organism (%)*		*P*
	The traditional testing (*n* = 151)	The mPCR testing (*n* = 68)	
Details of organisms detection			
Bacterial culture			
*S. pneumoniae*	13 (20%)	6 (19%)	0.884
*S. aureus*	9 (14%)	7 (21%)	0.479
*H. influenzae*	12 (18%)	4 (13%)	0.583
*klebsiella pneumoniae*	9 (14%)	4 (13%)	0.821
*Escherichia coli*	2 (3%)	1 (3%)	0.738
*Pseudomonas aeruginosa*	8 (12%)	2 (6%)	0.236
*Acinetobacter baumannii*	6 (9%)	3 (9%)	0.921
Total typical bacteria	65	32	0.685
Virological studies^†^			
Respiratory syncytial virus	33 (43%)	17 (45%)	0.546
Influenza A and B	32 (42%)	19 (30%)	0.291
Adenoviridae	5 (6%)	3 (8%)	0.718
Parainfluenza viruses 1–4	7 (9%)	2 (5%)	0.295
Human metapneumovirus	ND	1 (3%)	–
Rhinovirus/enterovirus	ND	1 (3%)	–
Coronavirus HKU1, NL63, 229E, OC43	ND	3 (8%)	–
Total viruses	77	38	0.600
Atypical agents			
*Mycoplasma pneumoniae*	22 (15%)	11 (17%)	0.703
Types of infection			
Virus infection only	25 (17%)	9 (13%)	0.642
Single virus	19 (13%)	5 (7%)	0.327
Multiplex viruses	6 (4%)	4 (6%)	0.548
Bacterium infection only	25 (17%)	12 (18%)	0.779
Single bacterium	15 (10%)	7 (10%)	0.792
Multiplex bacteria	10 (7%)	7 (10%)	0.463
Virus/bacterium coinfection	44 (29%)	21 (31%)	0.962

Data are no. (%) of patients

*ND* not done

*For typical bacteria and viruses, the percentage is of total isolates of each organism within the microbiological category and testing group. For *Mycoplasma*, the percentage is of infections detected in patients within the testing group

^†^Virological studies were diagnosed by serology or virus isolation in the traditional testing group, and were diagnosed by multiplex polymerase chain reaction PCR (mPCR) detection in the mPCR testing group

Initial antibiotic therapy at PICU admission was 56.6% (124/219), of which 86/151 (57.0%) was determined by traditional testing method and 38/68 (55.9%) by mPCR testing method. In patients with mPCR diagnosed with viral infection, initial antibiotic use (38%) was lower than that of the traditional testing group (78%) (*P* = 0.023). This was especially true of cephalosporin use, which reduced from 33% (traditional group) to 6% (mPCR group) (*P* = 0.043). Use of one antibiotic reduced from 37 to 19%, and use of two antibiotics reduced from 33 to 13%, but without statistical significance between the traditional and mPCR testing groups. Antibiotic treatment was similar between the two groups with bacterial infection and viral/bacterial coinfection.

No significant differences were observed in the percent of invasive mechanical ventilation used, length of hospital stay, and PICU stay between the traditional and mPCR testing groups. However, in patients with only viral infection or viral/bacterial coinfection, duration of mechanical ventilation was 5 days (range 3–7 days) and 12 days (range 4–16 days) in the traditional testing group, and it decreased to 3 days (range 1–5) and 7 days (range 3–9) in the mPCR testing group.

Although several observational studies have shown that mPCR detection of respiratory viruses has an impact on reducing the use of antibiotics in patients with viral pneumonia, there are fewer reports on children under 5 years of age [4–6]. In our study, most children were under 5 years of age and were discharged with a primary diagnosis of severe CAP. Similarly, the mPCR testing method has slightly increased the capability of identifying pathogens and has reduced initial antibiotic prescribed among children with viral infection [5, 6]. Pathogen-directed therapy can avoid unnecessary antibiotic or antiviral use, can facilitate more timely and effective use of drugs, and can help to prevent the secondary spread of infection, all of which contribute to shorten the length of hospitalization and to significantly influence patient management and disease prognosis [7].

Our study revealed that the mPCR testing method was associated with a significantly decreased duration of mechanical ventilation in patients with viral infection or viral/bacterial coinfection. Specifically, patients with bacterial infections tended to have a more severe course according to PICU admission rates, respiratory support necessity, clinical disease severity scales and length of hospitalization [8]. Previous research shows that the occurrence of pathogens with high antibiotic resistance in the lower respiratory tract increases with increased duration of hospital care and mechanical ventilation [9]. Alternatively, knowledge of a reduced duration of ventilation can be used to justify a prospective trial to assess the proper employment of antibiotics in children with severe CAP requiring invasive ventilation [10].

In the future, greater use of PCR amplification of bacteria may be accompanied by precise antibiotic prescription and can provide comprehensive information related to etiology in children with severe CAP.

## Data Availability

The datasets generated during and/or analyzed during the current study are available from the corresponding author on reasonable request.
